# GPR-160 Receptor Signaling in the Dorsal Vagal Complex of Male Rats Modulates Meal Microstructure and CART-Mediated Hypophagia

**DOI:** 10.3390/nu15102268

**Published:** 2023-05-11

**Authors:** Marcos J. Sanchez-Navarro, Tito Borner, Benjamin C. Reiner, Richard C. Crist, Willis K. Samson, Gina L. C. Yosten, Lauren Stein, Matthew R. Hayes

**Affiliations:** 1 Department of Psychiatry, Perelman School of Medicine, University of Pennsylvania, Philadelphia, PA 19104, USAcrist@pennmedicine.upenn.edu (R.C.C.); lmstein22@gmail.com (L.S.); 2 Department of Pharmacology and Physiology, Saint Louis University School of Medicine, 1402 S. Grand Boulevard, Saint Louis, MO 63104, USA

**Keywords:** GPR-160, CART, dorsal vagal complex, feeding, microglia

## Abstract

The g-protein coupled receptor GPR-160, recently identified as a putative receptor for the cocaine and amphetamine-regulated transcript (CART) peptide, shows abundant expression in the energy-balance control nuclei, including the dorsal vagal complex (DVC). However, its physiological role in the control of food intake has yet to be fully explored. Here, we performed a virally mediated, targeted knockdown (KD) of *Gpr160* in the DVC of male rats to evaluate its physiological role in control of feeding. Our results indicate that DVC *Gpr160* KD affects meal microstructure. Specifically, DVC *Gpr160* KD animals consumed more frequent, but shorter meals during the dark phase and showed decreased caloric intake and duration of meals during the light phase. Cumulatively, however, these bidirectional effects on feeding resulted in no difference in body weight gain. We next tested the role of DVC GPR-160 in mediating the anorexigenic effects of exogenous CART. Our results show that DVC *Gpr160* KD partially attenuates CART’s anorexigenic effects. To further characterize *Gpr160*+ cells in the DVC, we utilized single-nucleus RNA sequencing data to uncover abundant GPR-160 expression in DVC microglia and only minimal expression in neurons. Altogether, our results suggest that DVC CART signaling may be mediated by *Gpr160*+ microglia, which in turn may be modulating DVC neuronal activity to control food intake.

## 1. Introduction

The cocaine and amphetamine-regulated transcript (CART) is a neuropeptide produced in many brain areas, including nuclei involved in the regulation of energy balance, and accordingly, it modulates many physiological processes including feeding and energy expenditure [1]. While select reports suggest that CART signaling in the hypothalamus may have orexigenic effects [2,3,4,5,6], many groups have shown that CART signaling in the hindbrain, specifically in the nucleus tractus solitarius (NTS), has a potent anorectic effect [7,8,9,10,11,12,13,14,15,16]. These observations have been strengthened by rodent genetic models showing that animals that lack CART signaling have increased body weight [17,18,19]. Furthermore, several studies in humans have identified polymorphisms in the *Cartpt* gene that increase predisposition to obesity, anxiety, and depression [20,21,22,23,24,25,26]. Accordingly, the CART system represents a promising target for the development of novel pharmacotherapies to treat obesity. However, further knowledge on the mechanism of CART signaling is needed to fully understand this system’s complex effects on behavior.

Until recently, a major gap in our ability to exploit CART as a pharmacological target to treat disease was the absence of an identified CART receptor and genetic tools to target it. However, Yosten and colleagues [27] recently demonstrated that CART shows physical interaction with a g-protein coupled receptor (GPCR) known as GPR-160. Consistent with early studies looking at the intracellular changes in response to CART signaling [28,29], CART-mediated activation of GPR-160 leads to ERK phosphorylation. *Gpr160* has also been shown to be widely expressed in the brain [13], including many nuclei that show neuronal activation and physiological and behavioral responses following intraparenchymal CART administration, such as the hypothalamus and NTS. These results suggest that GPR-160 is a receptor for CART; however, a systematic and focused characterization of the acute and chronic role of brainstem GPR-160 in control of ingestive behavior has not been performed. 

In a recent study, Haddock et al. showed that passive immunoneutralization of GPR-160 attenuated CART’s anorexigenic effects as early as 3 h post injection [13]. This study served as a first pass evaluation of the role of GPR-160 in mediating CART’s anorexigenic effects. However, due to the nature of the approach, considerations must be made while interpreting the data. Most notably, it is possible that the GPR-160 antibody used may interact with epitopes shared with other GPCRs. Additionally, fourth cerebroventricular (4 V) passive immunoneutralization does not allow for the evaluation of GPR-160 signaling within distinct nuclei. Therefore, to more precisely evaluate the role of GPR-160 in mediating the well-described behavioral (i.e., hypophagia) and physiological responses (i.e., hypothermia and reduced locomotion) to hindbrain CART signaling, we developed an AAV-miRNA construct to knockdown *Gpr160* expression in the dorsal vagal complex (DVC; collectively comprised of the NTS, area postrema, and dorsal motor nucleus of the vagus). With this viral strategy we then evaluated the role of endogenous GPR-160 signaling in the control of body weight regulation, meal microstructure and food intake following a fasting period. We then tested whether CART’s anorectic, locomotor, and thermoregulatory effects were mediated by CART-induced DVC GPR-160 signaling. Lastly, using single nuclei RNA (snRNA) sequencing, we identified and began to phenotype the cells expressing *Cartpt* and *Gpr160* transcripts within the DVC, offering intriguing opportunities for future research. Altogether, our results further characterize the functional role of the recently deorphanized GPR-160 as a CART receptor in the control of ingestive behavior.

## 2. Materials and Methods

Animals and Drugs. All experiments were performed using male Sprague Dawley rats (300–325 g upon arrival; Charles River, Wilmington, MA, USA). Rats were individually housed (12 h light/dark cycle), provided undisturbed water access, and ad libitum fed a chow diet (Laboratory Rodent Diet 5001, Animal Specialties and Provisions), in cages equipped with BioDAQ food-intake monitoring systems (Research Diets Inc., New Brunswick, NJ 08901, USA) that measure episodic ad libitum feeding in the animal’s homecage. All procedures performed were approved by the Institutional Animal Care and Use Committee at the University of Pennsylvania and were performed following guidelines form the National Institute of Health. The cocaine- and amphetamine-regulated transcript peptide (2 μg/μL CART (55–102); Phoenix Pharmaceuticals, Burlingame, CA, USA) was dissolved in artificial CSF (aCSF; Harvard Apparatus, Holliston, MA, USA) for central injections.

Stereotaxic Surgery. Animals were split into two body weight matched groups (AAV1-GFP, *n* = 8; AAV1- *Gpr160* KD, *n* = 10) after which they were deeply anesthetized using a xylazine (2.7 mg/kg), ketamine (90 mg/kg), and acepromazine (0.64 mg/kg; IM) cocktail (KAX). They were then secured to the stereotaxic frame with the head in a ventroflexed position. A midline incision was then performed at the atlanto-occipital joint and the muscles were retracted to facilitate the opening of the joint capsule [30]. Bilateral AAV-stereotaxic injections of AAV1-CB7.CI.miR-*Gpr160*-eGFP.WPRE.bGH (200 nL, 200 nL/min, 1.632 × 10^13^ GC/mL, Penn Vector Core Gene Therapy Program) or AAV1-CAG-GFP (200 nL, 200 nL/min, 7 × 1012, Addgene) were applied using a 10 μL glass syringe (Nanofil, World Precision Instruments, Sarasota, FL 34240, USA) with a 33G beveled needle attached to a stereotaxic arm equipped with a microinfusion pump (World Precision Instruments, Sarasota, FL 34240, USA). AAV-stereotaxic injections were targeted to the DVC using the following coordinates from obex: ±0.4 M/L, +0.4 A/P, −0.4 D/V. Following AAV injections, a guide cannula was targeted to the 4th ventricle (+2.5 A/P, −5.2 D/V; from occipital suture) and secured with bone screws and dental cement. Analgesics were administered post-surgery (Meloxicam; 2 mg/kg) and rats were given a recovery period of 10 days prior to further testing or manipulation. Ten days post-surgery, cannula placement was verified for each animal by measuring the sympathoadrenal-mediated glycemic response to 4th ICV 5-thio-D-glucose (5TG, Santa Cruz Biotechnology; 210 μg in 2 μL of aCSF). Only rats that showed at least a doubling of blood glucose after 4th ventricle 5TG injections were included in subsequent testing. For the rats in the CART dose response study (*n* = 9), surgery, postoperative care and cannula placement validation was performed as described above, but these animals did not receive any viral treatment. 

General Procedures. Upon recovery, all rats were single housed in BioDAQ Episodic Intake Monitor-equipped cages in a 12 h light/dark cycle. The BioDAQ Episodic Intake Monitor collects moment-to-moment, undisturbed measurements of food intake at a very high resolution while animals are fed ad libitum (Rodent Diet 5001, LabDiets), making measurements of meal microstructure possible. The food intake data collected by the BioDAQ system is in the form of “bouts” and “meals”. Bouts were defined as uninterrupted feeding events of any time duration that ended after a period of inactivity >5 s, after which the next feeding event would be considered another discrete bout. A meal was defined as a collection of bouts that exceeded a total of 0.2 g or more and ended when the period of inactivity between 2 bouts was >900 s (15 min) [31]. The use of these previously established parameters for describing feeding microstructure facilitated the capture of differences in normal energy control induced by the virally mediated DVC *Gpr160* KD employed in this study. Food intake data were collected and analyzed for the following aspects of feeding microstructure: meal frequency (meal number/period), meal size (g/meal), and meal duration (min/meal), among others. All these parameters analyzed were calculated by the software provided by the manufacturer (BioDAQ Data Viewer software version 2.3.02).

Meal Microstructure Analysis. The following parameters were analyzed daily for each rat for 4 weeks post-viral injection using the BioDAQ Episodic Intake Monitor: meal frequency (number/period), meal size (g/meal), meal duration (min/meal). Daily body weight changes were also monitored every 24 h for the duration of the study. However, successful viral transduction (DVC *Gpr160* KD) was only assumed 2 weeks post viral delivery; therefore, food intake and body weight changes as a result of the viral knockdown was evaluated from weeks 2 to 4 post-surgery. During this period all animals remained undisturbed except for 1 h prior to the onset of the dark cycle during which general maintenance and daily body weight collection occurred.

Fasting-Induced Refeeding. Following the completion of the meal microstructure study, animals were food deprived for 24 h. Food was then reintroduced, and food intake data during the following 24 h of ad libitum food access was collected using the BioDAQ Episodic Intake Monitor system. Additionally, body weight loss in response to food deprivation, as well as weight gain in response to refeeding, were calculated. All animals were then allowed to return to baseline body weight before any further experiments were conducted.

DVC *Gpr160* KD Effects on CART Mediated Food Intake Suppression. Next, to assess the role of DVC GPR-160 in the control of 4th ventricle CART-mediated food intake suppression, animals were food deprived 2 h prior to dark cycle onset and CART (2 μg; 2 μg/μL) or aCSF was delivered into the 4th ventricle immediately before the onset of the dark cycle. Food was re-introduced at the onset of the dark cycle and cumulative food intake was measured for the next 24 h using the BioDAQ Episodic Intake Monitor system. Body weight was collected at 24 and 48 h post injection. The experiment was performed using a within-subject design and each treatment was spaced by at least 72 h. The CART dose was selected based on published work and preliminary tests in our lab to induce robust food intake suppression.

DVC *Gpr160* KD Effects on CART Mediated Hypothermia and Locomotion Effects. All animals were implanted with a PTD 4000 E-Mitter (STARR Life Sciences Corp., Oakmont, PA, USA) device in the abdominal cavity. Using this small, externally powered device, we collected continuous locomotion as well as changes in core body temperature for the duration of the experiment. This allowed us to assess the role of DVC GPR-160 in the control of 4th ventricle CART-mediated thermoregulation and locomotor effects. To do this, animals received CART or aCSF injection in the 4th ventricle immediately before the onset of the dark cycle and were undisturbed for the next 24 h during which continuous locomotor and body temperature data were collected. The experiment was performed using a within-subject design and each treatment was spaced by at least 72 h.

DVC Single Nuclei RNAseq Analysis. Data from our prior studies were used to phenotype DVC cell populations relevant to this study (NCBI GEO GSE 167981). Briefly, nuclei were isolated from frozen Sprague Dawley AP and NTS punches as previously described [32,33]. Nuclei capture and library preparation was performed at the Children’s Hospital of Philadelphia Center for Applied Genomics using the 10× Genomics Chromium Single Cell 3′ GEM, Library and Gel Bead Kit v3.1. Libraries were sequenced on an Illumina NovaSeq 6000 per manufacturer’s instructions. Sequencing reads were aligned to the rat pre-mRNA transcriptome (Rnor 6.0.101) using Cell Ranger v3.1 and the resulting filtered count matrices for all samples were merged in Seurat v3.1. Quality control, normalization, scaling, and clustering of these single nuclei RNAseq data have been previously described [32,34]. The following markers were used to annotate cell clusters: Microglia:—Cx3cr1; Endothelial—Cldn5; Astrocytes—Cldn10; Oligodendrocyte Precursor Cells—Pcdh15; Oligodendrocytes—Mag; Excitatory Neurons—Slc17a6; Inhibitory Neurons—Gad1; Neurons—Snap25; Tanycytes—Vim; Ependymocytes—Cfap52, Vim; Radial Glia—Notch2, Slc1a3.

Viral Knockdown Validation Using Fluorescent In-Situ Hybridization (FISH). Following completion of experiments, all animals were deeply sedated using the same KAX cocktail described for surgery, and decapitated. Brains were then quickly dissected, flash frozen in −70 °C isopentane, and stored at −80 °C. Each brain was then mounted on a cryostat (Leica CM3050s) with which 10 μm thick coronal sections at the level of the DVC were collected by directly mounting them onto Superfrost Plus slides. Then, *Gpr160* and GFP mRNA transcripts were detected using the RNAscope Multiplex Fluorescent Reagent Kit V1 following the manufacturer’s protocol. Briefly, slide mounted DVC coronal sections were fixed in pre-chilled 10%NBF for 15 min at 4 °C after which a series of dehydration washes were performed (5 min at room temperature in each of the following: 50%, 70%, 100%, 100% EtOH). All slides were then air dried and a hydrophobic barrier was drawn around all slices using a hydrophobic pen (Vector Labs, Newark, CA 94560, USA). The slides were then pretreated with Protease IV for 30 min at room temperature followed by two 2 min PBS washes. During the Protease IV pretreatment, a cocktail containing ACD-designed probes to detect the presence of *Gpr160* mRNA (Rn- *Gpr160*-C3, rat, REF: 515121-C3), as well as GFP mRNA (EGFP, REF: 400281) was prepared. This cocktail was added to all pretreated slides after which they were incubated for 2 h at 40 °C in a HybEZTM oven (ACD). All slides were then rinsed using manufacturer provided RNAscope wash buffer (Cat. 310091, ACD) and a series of amplification steps were performed using the Fluorescent Multiplex Detection Reagent kit (Cat. 320851, ACD) at 40 °C. Each step was separated by two 2 min washes with RNAscope wash buffer: 30 min AMP1-FL, 15 min AMP2-FL, 30 min AMP3-FL, and 15 min AMP4 Alt A-FL (C1, Alexa 488 nm; C2, Atto 550; C3, Atto 647). Following a final wash, slides were counterstained, and cover slipped using Fluorogel Mounting media with DAPI (Fisher Scientific, Waltham, MA, USA). Each slide was then imaged at 40× magnification using a BZ-X800 Fluorescent microscope (Keyence, Itasca, IL 60143, USA) and the FISH-IF module of the HALO-AI software was used to quantify the number of detected *Gpr160* transcripts in the images collected.

**Figure 1:**
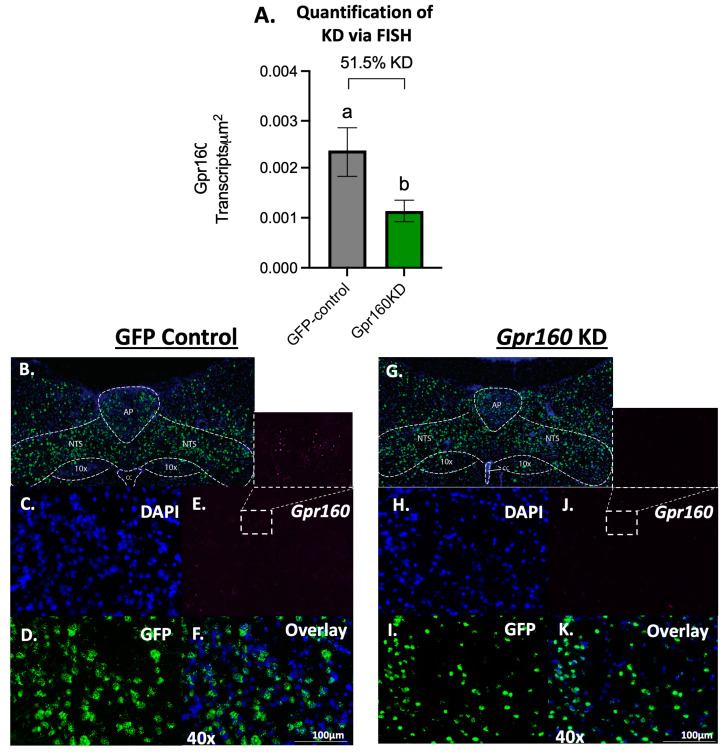
The viral knockdown strategy used generates substantial DVC GPR-160 KD as validated via fluorescent in-situ hybridization. (**A**) Fluorescent in-situ hybridization (FISH) was performed on DVC sections of all rats included in the behavioral study (AAV-GFP control *n* = 8; AAV- *Gpr160* KD *n* = 10) and the amount of *Gpr160* transcript per unit area was calculated. Data are expressed as ±SEM and analyzed with unpaired *t*-test. Significant differences between means are denoted by different letters (*p* < 0.05). (**B**–**F**) Representative DVC images of an AAV-GFP control treated animal following FISH. Scale bar 100 µm. (**G**–**K**) Representative DVC images of an AAV- *Gpr160* KD treated animal following FISH.

**Figure 2:**
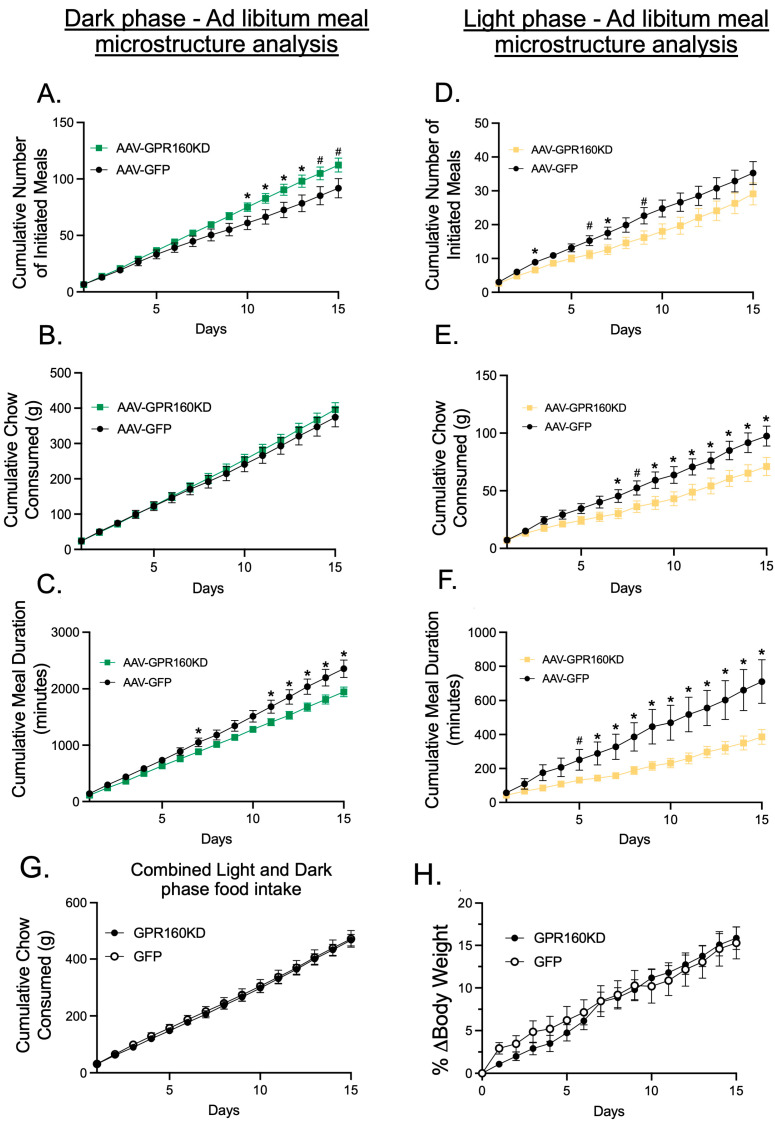
DVC *Gpr160* KD causes changes in dark and light phase meal microstructure without affecting total 24 h feeding. Rats (AAV-GFP control *n* = 8; AAV- *Gpr160* KD *n* = 10) were housed in BioDAQ cages equipped with the Episodic Intake Monitor and continuous recording of the meals taken by each animal were collected for 15 days starting two weeks after delivering the virus into the DVC. Data was split into light and dark phase to evaluate changes in meal microstructure. (**A**,**D**) DVC *Gpr160* KD produces an increase in the number of initiated meals during the dark phase and a slight decrease in the light phase. (**B**,**E**) DVC *Gpr160* KD reduces total light phase feeding without affecting total feeding during the dark phase. (**C**,**F**) DVC *Gpr160* KD reduces the time spent during a meal in both the light and dark phases. (**G**) *Gpr160* KD does not alter total food intake throughout a 24 h period. (**H**) *Gpr160* KD animals show no difference in body weight acquisition when compared to GFP treated controls. Data were analyzed using independent *t*-tests for each day and are expressed as ±SEM. (* = *p* < 0.05; # = *p* < 0.06).

**Figure 3:**
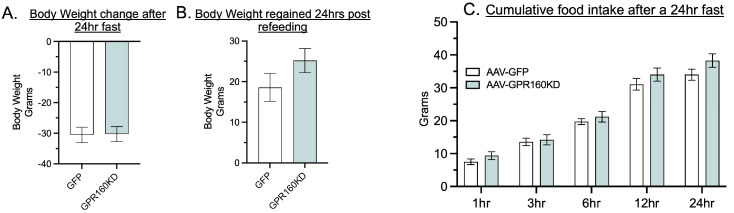
DVC *Gpr160* KD has no effect on food intake following a 24 h fasting period. (**A**–**C**) Rats (AAV-GFP control *n* = 8; AAV- *Gpr160* KD *n* = 10) fasted for 24 h after which food was reintroduced, food intake was measured for the next 1-, 3-, 6-, 12- and 24 h intervals, and body weight changes were assessed. Data are expressed as ±SEM and analyzed using 2-way ANOVA followed by Šidák post hoc test. No significant differences between means were identified (*p* < 0.05).

**Figure 4:**
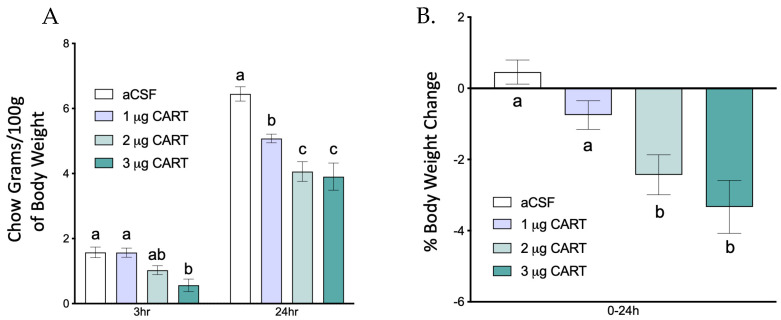
CART injections in the 4th ventricle dose dependently reduce food intake. (**A**) CART injection into the 4th ventricle results in dose dependent suppression of food intake over a period of 24 h (*n* = 9). (**B**) This treatment also results in dose dependent weight loss. Data are expressed as ±SEM and analyzed using 2-way ANOVA followed by Tukey post hoc test. Significant differences between means are denoted by different letters (*p* < 0.05).

**Figure 5:**
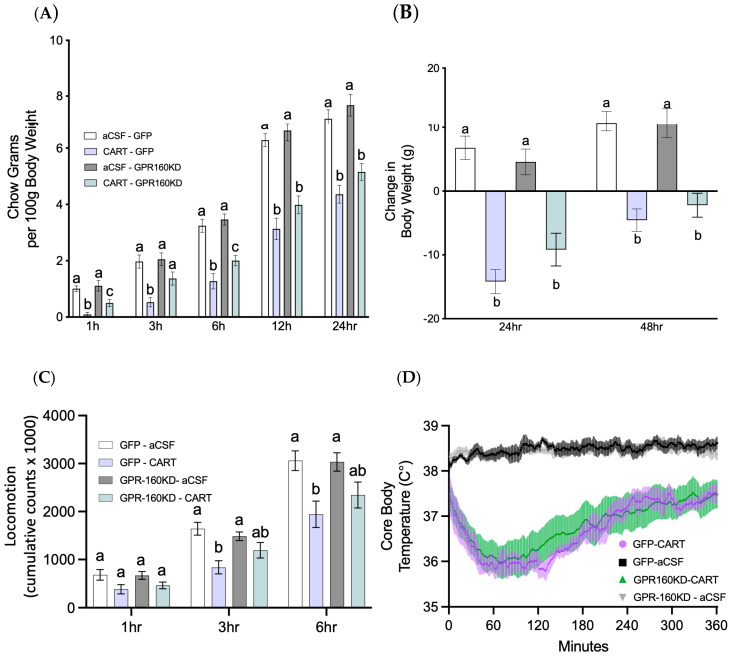
DVC *Gpr160* KD attenuates 4th icv CART mediated anorexigenic, but not locomotor or core body temperature effects. (**A**,**B**) Rats (AAV1-GFP control *n* = 8; AAV- *Gpr160* KD *n* = 10) were administered 4th icv CART at the onset of the dark cycle and food intake and BW was assessed at the following timepoints 1-, 3-, 6-, 12- and 24-h. (**C**,**D**) During this time, physiological effects such as locomotion and core body temperature changes were also measured. Data are expressed as ±SEM and analyzed using 2-way ANOVA followed by Student–Newman–Keuls post hoc test. Significant differences between means are denoted by different letters (*p* < 0.05).

Statistical Analysis. All data is shown as mean ± SEM with an α level set to *p* ≤ 0.05 for all experiments; all statistical analyses were completed using GraphPad Prism. For all datasets in Figure 1 and Figure 2, data were analyzed using independent *t*-tests. For all datasets in Figure 3, Figure 4 and Figure 5 where food intake and body weight were collected at multiple timepoints, data were analyzed using repeated measures ANOVAs that accounted for the within-subject experimental design while at the same time evaluating between-subject effects (drug treatment and AAV condition). Locomotion experiment data were similarly binned into timepoints of interests and analyzed as described for food intake experiments. Core body temperature data were analyzed by calculating area under the curve and using repeated measures ANOVA to evaluate within-subject as well as between-subject effects. The post hoc tests used for Figure 3, Figure 4 and Figure 5 were Šidák, Tukey and Student–Newman–Keuls, respectively. Lastly, the difference in *Gpr160* expression between AAV-GFP control and AAV- *Gpr160* KD animals was analyzed using an unpaired *t*-test. For all figures, significant differences between means are denoted by different letters (*p* < 0.05). 

## 3. Results

### 3.1. Endogenous DVC GPR-160 Regulates Light and Dark Phase Meal Microstructure 

To determine whether endogenous signaling of the newly deorphanized CART receptor GPR-160 plays a physiological role in the control of daily food intake regulation, an AAV1 encoding a microRNA to knockdown *Gpr160* (AAV1- *Gpr160* KD) or an empty control vector was bilaterally injected directly into the DVC at the level of the AP (200 nL/hemisphere). When compared to empty control vector treated, AAV1- *Gpr160* KD treated animals show a 51.5% reduction in DVC *Gpr160* transcripts detected when quantified via Fluorescent In-Situ Hybridization (FISH; Figure 1). 

Daily food intake data was subdivided into light and dark phase feeding to capture potential roles of endogenous GPR-160 signaling at different times of the day. During the dark phase, DVC *Gpr160* KD animals show decreased meal duration and increased number of initiated meals with no effect in cumulative food intake when compared to controls (Figure 2A–C). Conversely, during the light phase, knockdown animals show a decrease in cumulative food intake, meal duration, and a decrease in the number of initiated meals only in days 3 and 7 of the experiment (Figure 2D–F). It is worth noting that, although knockdown animals showed decreased cumulative food intake during the light phase, there was no difference in the total amount of food consumed when light and dark phases were analyzed together (Figure 2G), and knockdown animals showed no difference on body weight gain when compared to controls (Figure 2H). These results suggest that endogenous DVC GPR-160 signaling regulates different aspects of meal microstructure at different times of the day without controlling the total amount of food an individual consumes in a 24 h period.

### 3.2. DVC Gpr160 KD Does Not Affect Food Intake in Response to a 24-h Fast

Vagal afferent neurons (VAN) terminating in the mNTS have been shown to produce CART that is released into the mNTS and is required for satiation [16]. Furthermore, VAN *Cartpt* expression has been shown to decrease during fasting and increase following refeeding [16]. Therefore, we hypothesized that DVC GPR-160 CART-mediated endogenous signaling could be regulating the amount of food consumed following a period of fasting. However, our data show that knocking down *Gpr160* in the DVC has no effect on the amount of food consumed following a 24 h fasting period, as well as body weight regain 24 h after food is reintroduced (Figure 3A–C). These data suggest that endogenous signaling of DVC GPR-160 is not necessary for the control of meal size or termination after a fasting period.

### 3.3. DVC Gpr160 KD Attenuates 4th ICV CART-Mediated Food Intake without Affecting Locomotor and Thermoregulatory Effects

To identify an effective 4th ventricular dose, a cohort of rats (*n* = 9) received a 4th ICV injection of three different doses of CART (1, 2 or 3 μg/μL) just before lights out. Food intake was then measured at an early (3 h) and late (24 h) timepoint, and 24 h change in body weight was collected. Injection of CART into the 4th ICV dose dependently decreased food intake and body weight (Figure 4A,B). However, high doses of CART delivered intracerebroventricularly have been reported to produce seizure-like events in rats [12,35]. Consistent with this, the 3 μg/μL dose tested here also produced tremors and seizure-like events. Therefore, further experiments were performed using a 2 μg/μL CART dose since it did not cause such side effects.

CART has been shown to be a ligand for the g-protein coupled receptor GPR-160 [13]; however, whether the well-established anorexigenic, hypothermic and locomotor effects of 4th ICV CART delivery are mediated by this receptor in the DVC has not been fully investigated. Here, we used a viral knockdown approach to test whether DVC GPR-160 is necessary for 4th ICV CART’s food intake reducing effects. Our data show that DVC *Gpr160* knockdown attenuates CART’s early anorexigenic effects. As shown in Figure 5A, *Gpr160* KD animals demonstrated an attenuation of CART’s anorexigenic effects at 1-, 3-, and 6-h post CART delivery when compared to controls. However, this attenuation is not observed at later timepoints in a 24-h period, resulting in no attenuation of body weight loss in response to CART (Figure 5B). These results suggest that CART-mediated GPR-160 signaling is responsible for the early anorexigenic action of CART signaling in the hindbrain. 

Previous work has shown that hindbrain CART injections reduce locomotion as well as core body temperature [7]. However, whether these CART-induced changes are due to CART signaling via DVC GPR-160 has not been evaluated. To test this, all animals received 4th ICV CART injections as previously described, after which locomotion and core body temperature data were collected. Our data show that DVC *Gpr160* knockdown failed to attenuate the CART-mediated decrease in locomotion and core body temperature (Figure 5C,D). Together, these data suggest that CART-mediated activation of DVC GPR-160 is exclusively responsible for CART’s early anorexigenic effects, but not CART’s previously described hypothermic and locomotor effects. 

### 3.4. DVC Single Nucleus RNAseq Data Reveals Substantial Gpr160 Expression in Microglia, but Not Neurons

**Figure 6:**
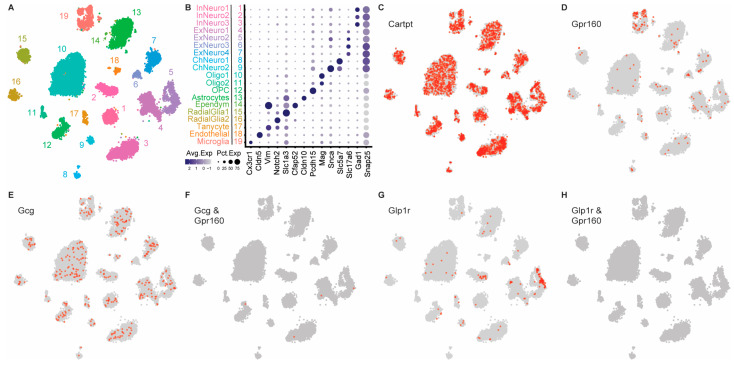
snRNAseq analysis of *Gpr160* expression in DVC. A tsne (**A**) and dotplot (**B**) identifying the major cell types in the rat AP and NTS color-coded by cluster. Gene expression was used to annotate major cell types and the size and color of dots are proportional to the percentage of cells expressing the gene (Pct.Exp) and the average expression level of the gene (Avg.Exp), respectively. (**C**–**H**) Highlighted tsne plots identifying nuclei containing transcripts for the identified gene(s). While expression of *Cartpt* was ubiquitous, *Gpr160* expression was predominantly observed in microglia, with negligible expression in *Gcg* and *Glp1r* expressing neuronal subtypes.

Due to the complexity and multifaceted nature of CART signaling, deciphering this system’s mechanism of action will require thorough knowledge of the different cell types that express transcripts for the CART receptor gene *Gpr160*. To this end, we utilized snRNA sequencing data of the DVC of sated rats fed a chow diet to identify relevant populations amongst DVC cell types (Figure 6A,B). Intriguingly, while *Cartpt* was expressed in many DVC cell types (Figure 6C), *Gpr160* is most abundantly expressed in DVC microglia, with only sparse expression in some neuronal cell types (Figure 6D). Importantly, our data also show that both preproglucagon (*Gcg*) expressing (Figure 6E,F) and *Glp1* receptor (*Glp1r*) expressing (Figure 6G,H) neurons do not express *Gpr160*. This finding is of particular relevance since previous work has shown that CART’s anorexigenic effects are attenuated by GLP-1R antagonism, suggesting that CART signaling is dependent on downstream GLP-1R signaling. Consequently, these data raise the possibility that CART may be modulating neuronal activity indirectly via action on microglia that then results in modulation of DVC neurons and feeding regulation. Although future experiments will be needed to fully understand the mechanism of action, our data further suggests that CART may be acting on *Gpr160*+ microglia which are in turn modulating either GLP-1 producing preproglucagon neurons and/or *Glp1r* expressing neurons to regulate feeding.

## 4. Discussion

Since its discovery in 1981, the role of CART signaling has been extensively studied in a multitude of brain nuclei. These studies highlight CART as a multifaceted neuropeptide with the ability to modulate multiple physiological processes, such as cardiometabolic functions, thermoregulation, motivated, and ingestive behaviors. Due to CART’s ability to potently reduce food intake when delivered into the hindbrain, the CART system represents a promising target for the development of pharmacotherapies to treat obesity. However, until recently, research into the mechanisms of CART action has been stalled by the absence of a CART receptor and consequently antagonists to target said receptor. Yosten and colleagues [27] recently identified and deorphanized the G-protein coupled receptor, GPR-160 as a CART receptor, setting the stage for deciphering the cellular substrates and signaling mechanisms mediating CART’s physiological effects. Here, using a novel viral strategy to chronically knockdown *Gpr160* transcripts in the DVC, along with analysis of DVC snRNA sequencing, we show that (1) DVC GPR-160 receptors mediate, at least part, of CART’s anorexigenic effects, (2) DVC GPR-160 endogenous signaling is important for the control of normal meal microstructure, and that (3) *Gpr160* is preferentially expressed in the DVC on microglia. These findings identify DVC GPR-160 as an important regulator of CART signaling in the context of food intake, suggesting that further understanding of GPR-160’s mechanism of action would make this system a promising target for future pharmacotherapies.

Following the identification of GPR-160 as a receptor for CART [27], Haddock et al. (2020) [13] began characterizing the role of GPR-160 in the control of hindbrain CART anorexigenic effects. In their study, they used a passive immunoneutralization approach to show that GPR-160 signaling modulates CART’s anorexigenic and antidipsogenic effects. However, the use of the passive immunoneutralization approach presents some limitations such as: (1) the antibody is being delivered into the 4V so the specific nuclei of action are not being identified, and (2) the manipulation might not be as specific as desired given that the antibody could be targeting other GPCRs of the same family as GPR-160. Consequently, to better determine whether the attenuation of CART’s effects is due to decreased GPR-160 signaling we developed an AAV knockdown strategy (miRNA). With this approach, we were able to selectively decrease *Gpr160* expression in the DVC and evaluate the contribution of DVC GPR-160 signaling on the control of CART’s anorexigenic effects. Consistent with Haddock et al. (2020) [13], we show that DVC GPR-160 signaling mediates CART’s ability to reduce food intake; however, our data shows that DVC *Gpr160* KD only attenuates CART’s early and not late satiation effects. This finding underscores the complexity of CART signaling and its role in the control of behavior and physiological processes, hinting at the possibility that other CART receptor(s) may exist. Our viral approach presents its own set of limitations that must be considered. Namely, the percent KD achieved via our viral KD (~51%) may have been insufficient to completely attenuate all the physiological and behavioral effects mediated by exogenous CART. It is also possible that some compensatory changes derived from microglia-expressing *Gpr160* occurred in response to *Gpr160* KD that blunted our ability to observe more robust behavioral and physiological phenotypes following *Gpr160* knockdown. 

One of the strengths of the viral approach employed here is that it enables the evaluation of the effect of chronic decreased DVC GPR-160 signaling. This provides insights into the role of endogenous CART signaling in the control of different aspects of food intake across longer periods of time. With this in mind, we evaluated the role of GPR-160’s endogenous signaling in the control of normal meal microstructure during the light and dark phase, as well as total 24-h consumption. We show that endogenous DVC GPR-160 signaling is important for regulating meal initiation and duration during the dark phase and meal duration and size during the light phase. Importantly, DVC *Gpr160* KD seems to have no effect on any of the meal microstructure parameters evaluated when the data for light and dark phases is combined into a 24 h period. These results show that DVC GPR-160 endogenous signaling regulates normal meal microstructure differently across a 24 h period, but has no effect on the total amount of food consumed. This conclusion is further strengthened by the fact that DVC *Gpr160* knockdown has no effect on body weight gain when compared to GFP controls. The CART peptide has been shown to have a diurnal rhythm in both the blood and the brain [36,37,38]. Diurnal variance of *Cartpt* and *Gpr160* expression has not been directly tested in DVC; however, the vagus nerve expresses *Cartpt* and vagal afferents are known to release CART into the NTS. Given that CART can cross the blood brain barrier and presumably activate the vagus nerve [39], one would expect vagal CART release into the NTS to show rhythmic patterns similar to those observed in the blood. Indeed, prior work has shown a quite clear role for rhythmic vagal afferent transmission of gastrointestinally derived satiation signals [40]. Although untested, this hypothesis could present a possible explanation for how our DVC *Gpr160* KD-mediated chronic decrease in GPR-160 signaling can modulate temporal feeding patterns throughout the 24 h period. Consequently, future experiments should directly test whether *Cartpt* expression shows rhythmic patterns in the DVC and the vagus nerve. Furthermore, these studies were performed on male rats fed a chow diet and future experiments will need to be repeated with female rats, as well as with animals fed an obesogenic high fat diet.

Understanding how hindbrain CART signaling produces anorexigenic effects will require further knowledge on the types of cells that express *Gpr160*, and therefore, have the ability to respond to CART. Consequently, we analyzed snRNA sequencing of the DVC and found that, although *Gpr160* is expressed at low levels in different types of neurons in the DVC, *Gpr160* is most robustly expressed in DVC microglia. These data suggest that DVC microglia may respond to CART via GPR-160 signaling and modulate neuronal activity to influence feeding behavior. Previous literature has implicated microglia in the control of energy homeostasis [41]; however, the mechanism by which this is possible has not been explored. As microglia have been shown to regulate neuronal activity via negative feedback control [42], it is interesting to consider the impact that CART activation of *Gpr160*+ microglia would have on specific neural ensembles in the DVC to control ingestive behavior. Future studies employing spatial transcriptomics to look at the microenviroment of neurons surrounding *Gpr160*+ microglia will undoubtedly shed light on these types of questions. Nonetheless, the current data, together with the limited reports on microglia-neuronal communication regarding energy balance, collectively suggest that DVC microglia may be responding to CART via GPR-160 signaling to affect the activity of an unknown neuron population(s) within the DVC to modulate ingestive behavior. Interestingly, although CART’s anorexigenic effects have been shown to be attenuated following GLP-1R antagonism [7,43], our data show that no *Gcg* or *Glp1r* expressing cell type in the DVC expresses *Gpr160*. These results further support the idea that CART may be acting on *Gpr160*+ microglia which are in turn modulating either *Gcg* expressing preproglucagon neurons and/or *Glp1r* expressing neurons to regulate feeding. One limitation of this study is that *Gpr160* expression in DVC cell types was only evaluated in sated animals fed a chow diet. Future experiments should evaluate whether differences in diet composition or energy state (sated vs. food deprived) can change the levels of *Gpr160* expression in the different identified DVC cell types.

A recent report was published that contradicts our initial finding regarding the association between GPR-160 and CART [44]. We acknowledge that it is possible, and perhaps even plausible, that GPR-160 is one of many potential receptors for CART; that GPR-160 is a co-receptor or part of a larger signalosome for CART; or that the affinity of GPR-160 for CART is dependent upon expression of a receptor modulating protein (i.e., biased agonism). Interestingly, the authors of this aforementioned report did not allow for any of these possibilities in their conclusions [44], in spite of several technical limitations of their studies (e.g., non-specific primers to detect *Gpr160* mRNA, failure to confirm membrane expression of transfected GPR-160, and others). Regardless, their study reiterates that there is yet much to learn regarding the biology of GPR-160 and CART, and future studies must address the identity of the full signalosome and intracellular cascades that mediate the cellular functions of CART.

Overall, this work shows that DVC GPR-160 signaling is at least partially responsible for CART’s well-established anorexigenic effects when delivered to the brainstem at pharmacological doses. This work also shows that DVC GPR-160 endogenous signaling regulates normal meal microstructure and begins to characterize the DVC cell types that express *Gpr160*. Together, these findings provide a framework for future experiments to further dissect this signaling pathway and assess the CART system’s potential as a target for the development of more effective pharmacotherapies. Achieving this will require the development of CART system-specific manipulations, such as GPR-160 antagonists and genetic models, that allow for more efficient manipulations of different neuronal/cell populations that express *Gpr160*. Having identified the elusive CART receptor, it will undoubtedly be a matter of time until these tools are readily available, making the identification of CART mechanisms of action more feasible than ever.

## Data Availability

The published article includes all data generated or analyzed during this study. No code was used or generated in this study.
